# Intrauterine adhesion in ultrasound-guided manual vacuum aspiration (USG-MVA) versus electric vacuum aspiration (EVA): a randomised controlled trial

**DOI:** 10.1186/s12884-024-06328-y

**Published:** 2024-02-14

**Authors:** Jacqueline Pui Wah Chung, Tracy Sze Man Law, Karen NG, Patricia Nga Ping IP, Tin Chiu Li

**Affiliations:** Department of Obstetrics and Gynaecology, The Chinese University of Hong Kong, Prince of Wales Hospital, Shatin, New Territories, Hong Kong SAR China

**Keywords:** First-trimester miscarriage, Surgical evacuation, Ultrasound-guided manual vacuum aspiration, Intrauterine adhesion

## Abstract

**Background:**

Intrauterine adhesion (IUA) can arise as a potential complication following uterine surgery, as the surgical procedure may damage the endometrial stratum basalis. The objective of this study was to assess and compare the occurrence of IUA in women who underwent ultrasound-guided manual vacuum aspiration (USG-MVA) versus electric vacuum aspiration (EVA) for managing first-trimester miscarriage.

**Methods:**

This was a prospective, single-centre, randomised controlled trial conducted at a university-affiliated tertiary hospital. Chinese women aged 18 years and above who had a delayed or incomplete miscarriage of ≤ 12 weeks of gestation were recruited in the Department of Obstetrics and Gynaecology at the Prince of Wales. Recruited participants received either USG-MVA or EVA for the management of their miscarriage and were invited for a hysteroscopic assessment to evaluate the incidence of IUA between 6 and 20 weeks after the surgery. Patients were contacted by phone at 6 months to assess their menstrual and reproductive outcomes.

**Results:**

303 patients underwent USG-MVA or EVA, of whom 152 were randomised to ‘USG-MVA’ and 151 patients to the ‘EVA’ group. Out of the USG-MVA group, 126 patients returned and completed the hysteroscopic assessment, while in the EVA group, 125 patients did the same. The incidence of intrauterine adhesion (IUA) was 19.0% (24/126) in the USG-MVA group and 32.0% (40/125) in the EVA group, showing a significant difference (*p* < 0.02) between the two groups. No significant difference in the menstrual outcomes at 6 months postoperatively between the two groups but more patients had miscarriages in the EVA group with IUA.

**Conclusions:**

IUAs are a possible complication of USG-MVA. However, USG-MVA is associated with a lower incidence of IUA postoperatively at 6–20 weeks. USG-MVA is a feasible, effective, and safe alternative surgical treatment with less IUA for the management of first-trimester miscarriage.

**Trial registration:**

The study was registered with the Centre for Clinical Research and Biostatics- Clinical Trials Registry (CCRBCTR), which is a partner registry of the WHO Primary Registry-Chinese Clinical Trials Registry (ChiCTR) (Unique Trial Number: ChiCTR1900023198 with the first trial registration date on 16/05/2019)

## Background

It is estimated that approximately one in four pregnant women may experience a miscarriage. When it comes to managing miscarriages, conservative, medical, or surgical approaches can be taken. In traditional surgical procedures, electric vacuum aspiration (EVA) is commonly used under general anaesthesia, sometimes followed by uterine curettage.

Introduced in the United States in 1973, manual vacuum aspiration (MVA) emerged as an alternative surgical technique for treating early pregnancy loss. It was developed as a replacement for the older method of dilation and sharp uterine curettage [1]. Manual vacuum aspiration (MVA) offers a non-electric alternative for the procedure, utilising a specially designed hand-held 60 ml charged syringe to generate the required suction force. With the aid of a flexible or rigid cannula attached to the syringe, the intrauterine contents can be effectively aspirated.

MVA provides a cost-effective, portable, user-friendly, and convenient option for treating early pregnancy loss [2]. It offers the advantage of not requiring general anaesthesia and can be performed without the use of electricity. Patients are given simple oral analgesics or conscious sedation before MVA. Studies have shown that MVA has high efficacy, with similar rates of successful evacuation compared to EVA [2, 3], and is associated with minimal complications [3–5]. Ultrasound guidance (USG) during MVA may reduce the discomfort during the introduction [5], shorten the procedure [5] and ensure that the evacuation process is complete [3]. Theoretically, it can reduce the formation of future intrauterine adhesion (IUA) as it helps reduce the damage to the endometrium stratum basalis. In our previous paper [5], USG-MVA is shown to be an effective, feasible and safe treatment option for the management of early pregnancy loss in an outpatient setting. The complete evacuation rate of USG-MVA has been reported to be as high as 97.1%, which is comparable to the rate reported for EVA (97.5%) in a previous systematic review [3]. 

IUA or Asherman’s syndrome may occur after uterine surgery for miscarriages as the endometrial stratum basalis maybe damaged during surgery. IUA can be asymptomatic or manifest as menstrual disturbances such as amenorrhea or hypoamenorrhea, dysmenorrhea, recurrent miscarriages, or infertility issues. The presence of IUA can have a negative impact on future fertility, as it can affect the successful implantation of embryos [6]. Moreover, IUA increases the rate of further miscarriages, potentially lead to abnormal placentation, fetal growth restriction, preterm delivery, and post-partum haemorrhage [7]. Timely identification of IUA is crucial, as prompt intervention can help prevent additional complications [8]. Hysteroscopy is considered the gold standard for diagnosing intrauterine adhesions (IUA).

Previous studies showed that the incidence of IUA after EVA could range from 7.7 − 38% [9–11]. However, there is limited data to evaluate the incidence of IUA after MVA for early miscarriages. The objective of this study was to investigate the incidence of IUA following the use of USG-MVA for managing first-trimester miscarriages and compare it to the incidence after EVA performed under general anaesthesia. We hypothesised that USG-MVA was associated with a lower rate of IUA when compared to EVA.

## Methods

This was a prospective single-centre, randomised controlled trial conducted in the Department of Obstetrics and Gynaecology of the Prince of Wales Hospital from May 2019 to September 2022.

### Study population

The study enrolled Chinese women aged 18 years or older, who were hemodynamically stable and had a delayed miscarriage of up to 12 weeks of gestation or an incomplete miscarriage. Recruitment took place at the Department of Obstetrics and Gynaecology of the Prince of Wales Hospital.

Women were excluded if (1) after examination it was considered that it was not feasible to perform the USG-MVA procedure due to cervical stenosis, fibroid uterus ≥ 12 weeks in size, uterine malformation or known bleeding disorder; (2) there was a clinical suspicion of active infection and inability to tolerate pelvic examination; and (3) history of allergy to misoprostol.

Missed miscarriage was defined as either (1) a lack of cardiac activity at crown rump length ≥ 5 mm; or (2) an intrauterine gestational sac with a mean sac diameter of ≥ 20 mm without a fetal pole; or (3) an intrauterine gestational sac ≤ 20 mm with no interval growth or persistent absence of fetal cardiac pulsation on rescanning 7–10 days later. Incomplete miscarriage was defined as the passage of products of conception with residual products on ultrasound (heterogenous intrauterine products or those with homogenous intra-uterine dimension measuring ≥ 11cm^2^ – sagittal and transverse plane) and/or if the patient had persisting symptoms (pain and or bleeding) [12]. The patients’ sociodemographic background, menstrual and reproductive history were recorded on a pre-defined datasheet.

### Randomisation

Randomisation was performed at a 1:1 ratio according to a computer-generated number list into two groups. After written informed consent was obtained from all the participants, a study nurse opened the opaque, sealed envelope containing the randomised number for each of them prior to surgery. The USG-MVA group or the EVA group as the control group was chosen randomly for all eligible patients who gave their consent for the study.

### USG-MVA group

Patients were randomly assigned to the USG-MVA group and admitted to the Gynaecological outpatient day ward for the procedure. The USG-MVA procedure was conducted as a day procedure in an outpatient setting, following a predefined protocol. Cervical priming was carried out by administering 400 mg of misoprostol orally to each patient, two to three hours before the USG-MVA procedure. In cases where there was heavy bleeding or the passage of products of conception, pelvic examination and/or ultrasound were performed to assess the amount of retained products and ensure that the USG-MVA procedure was still indicated. Routine observations such as blood pressure, pulse, and temperature were measured. All patients received 500 mg of naproxen orally one hour prior to the procedure. If a patient had an allergy to non-steroidal anti-inflammatory drugs, paracetamol or codeine was administered as an alternative. Patients were advised not to empty their bladder before the procedure.

USG-MVA was performed using a 60 ml charged syringe (MedGyn Aspiration Kit IV, 02511, 100 W Industrial Rd., Addison, IL 60,101 USA) with a flexible curette (size 4 to 7 mm, subject to doctor’s discretion) attached to it. An experienced doctor and a nurse performed the procedure together. Transabdominal USG during MVA was performed using the E730 Expert US system. Before the procedure, a paracervical block was performed using a 23-gauge dental needle syringe to inject 5 ml of 2% xylocaine into a depth of 0.5 cm at the cervical-vaginal juncture at 4, 5, 7, and 8 o’clock position to reach the uterosacral ligaments and 5 ml of local lidocaine gel (Xylocaine 2%) was applied to the cervix a few minutes before the insertion of the MVA catheter. Once the ultrasound showed that the uterine cavity was empty, the USG-MVA procedure was stopped.

### EVA group

Traditional EVA was performed in the operation theatre under general anaesthesia. The patient was admitted to the day surgical ward on the morning of the operation and kept fasting. After general anaesthesia, the patient would be placed in a lithotomy position. The procedure was performed without USG. A flexible curette (size 8, 10 or 12 mm, subject to doctor’s discretion) was used for the procedure. Once the surgeon could feel the uterus contracting against the suction catheter, the procedure would be stopped. Further systematic curettage with a sharp curette was performed to confirm complete uterine evacuation. Patients were then discharged two hours after USG-MVA and 6 h after EVA if they were clinically and haemodynamically stable with minimal bleeding and pain. Patients were then provided with a dedicated hotline and advised to contact the ward with any problems on a 24-hour basis.

### Outpatient hysteroscopy (OPH)

For patients who agreed to join the study, an outpatient hysteroscopy was performed within 6–20 weeks from their initial USG-MVA or EVA procedure by a designated team of experienced gynaecologists with similar surgical experience who were blinded to the women’s initial surgical procedure performed for the first-trimester miscarriage. Before OPH, a pregnancy test was performed and any recent coitus was recorded [6]. 

The outpatient hysteroscopy was performed using a 2.9 mm rigid diagnostic hysteroscopy (Karl Storz, Germany) under aseptic technique and the endometrial cavity was visualised and assessed systematically. Normal saline was used for uterine distension and no cervical dilatation was required. The patient was discharged from the hospital approximately one hour after the hysteroscopic assessment.

### Intrauterine adhesion assessment

The presence of any IUA, the status of bilateral ostia and endometrial cavity, and endometrium were assessed. The severity and nature of the IUA were classified using the American Fertility Society (AFS) classification of IUA [13]. There are multiple classification systems for IUA, including the classifications proposed by Hamou et al. [14], Donnez and Nisolle [15], and the European Society of Hysteroscopy (ESH) [16]. While the ESH classification is very comprehensive, it may have limited practicality in clinical practice due to its complexity. We have chosen the AFS classification system over other classifications due to its comprehensiveness and wide usage in classifying IUA. The AFS classification system incorporates clinical symptoms, such as menstrual pattern, as an indicator of severity. This is important for estimating the potential regeneration of the endometrium after adhesiolysis and serves as a key marker for determining the prognosis following treatment.

During the hysteroscopic procedure, if it is deemed feasible to remove the identified IUA in an outpatient setting, consent would be obtained prior to the hysteroscopic assessment. The adhesions would then be removed using hysteroscopic scissors [6]. 

### Subsequent menstrual and reproductive assessment

Six months following their initial USG-MVA or EVA, all women were contacted by phone to document the information about their menstrual histories, with or without the feeling on pelvic pain, and their reproductive outcomes. Hypomenorrhea, or lighter menstrual flow, has been associated with the presence of IUAs. In cases where IUAs are present, the formation of scar tissue can reduce the amount of normal endometrium available for shedding during menstruation [6]. Women were considered lost to follow-up if they remained uncontactable after 3 attempts.

### Outcome measures

The primary outcome was the incidence of IUA after USG-MVA and EVA as assessed by the outpatient hysteroscopy at 6–20 weeks from the surgery. Secondary outcomes were the rate of complications from the hysteroscopy assessment (including uncontrolled bleeding requiring intervention, infection and uterine perforation), the type and extent of the IUA, subsequent menstrual and reproductive outcomes at 6 months postoperatively.

### Sample size

According to a previous meta-analysis, the incidence of IUA was 18.5% after EVA. From a previous case series involving 262 office MVA, 5 cases of IUA were noted, giving an incidence of IUA of 1.9% [17]. 

With the incidence of IUA after EVA as 18.5% and assumption on the incidence of IUA after USG-MVA as 5%, a total of 115 subjects are required in each group with 90% power at a 5% 2-sided significant level. Allowing for a dropout rate of 30%, a total of 150 subjects are required in each group, 300 cases in total.

### Ethics approval

The study was approved by the Institutional Review Board of our institution (Joint Chinese University of Hong Kong- New Territories East Cluster Clinical Research Ethics Committee registration number CRE 2019.079. The study was registered with the Centre for Clinical Research and Biostatics- Clinical Trials Registry (CCRBCTR), which is a partner registry of the WHO Primary Registry-Chinese Clinical Trials Registry (ChiCTR) (Unique Trial Number: ChiCTR1900023198 with the first trial registration date on 16/05/2019).

### Statistical analysis

Data are summarised as medians (interquartile range (IQR)) for continuous variables and frequencies (%) for categorical variables. Comparison of baseline characteristics and study outcomes between the two surgical modalities were performed using the Mann-Whitney test for continuous variables and the chi-square test or Fisher’s exact test for categorical variables. All statistical tests were performed using SPSS 26.0 (IBM Corporation, New York) with a two-sided *p*-value < 0.05 considered statistically significant. Multivariate logistic regression analysis was performed to assess the association between the presence/absence of IUA and adjusted for confounding factors. All statistical tests were performed using SPSS 26.0 (IBM Corporation, New York) with a two-sided *p*-value < 0.05 considered statistically significant.

## Results

**Fig. 1:**
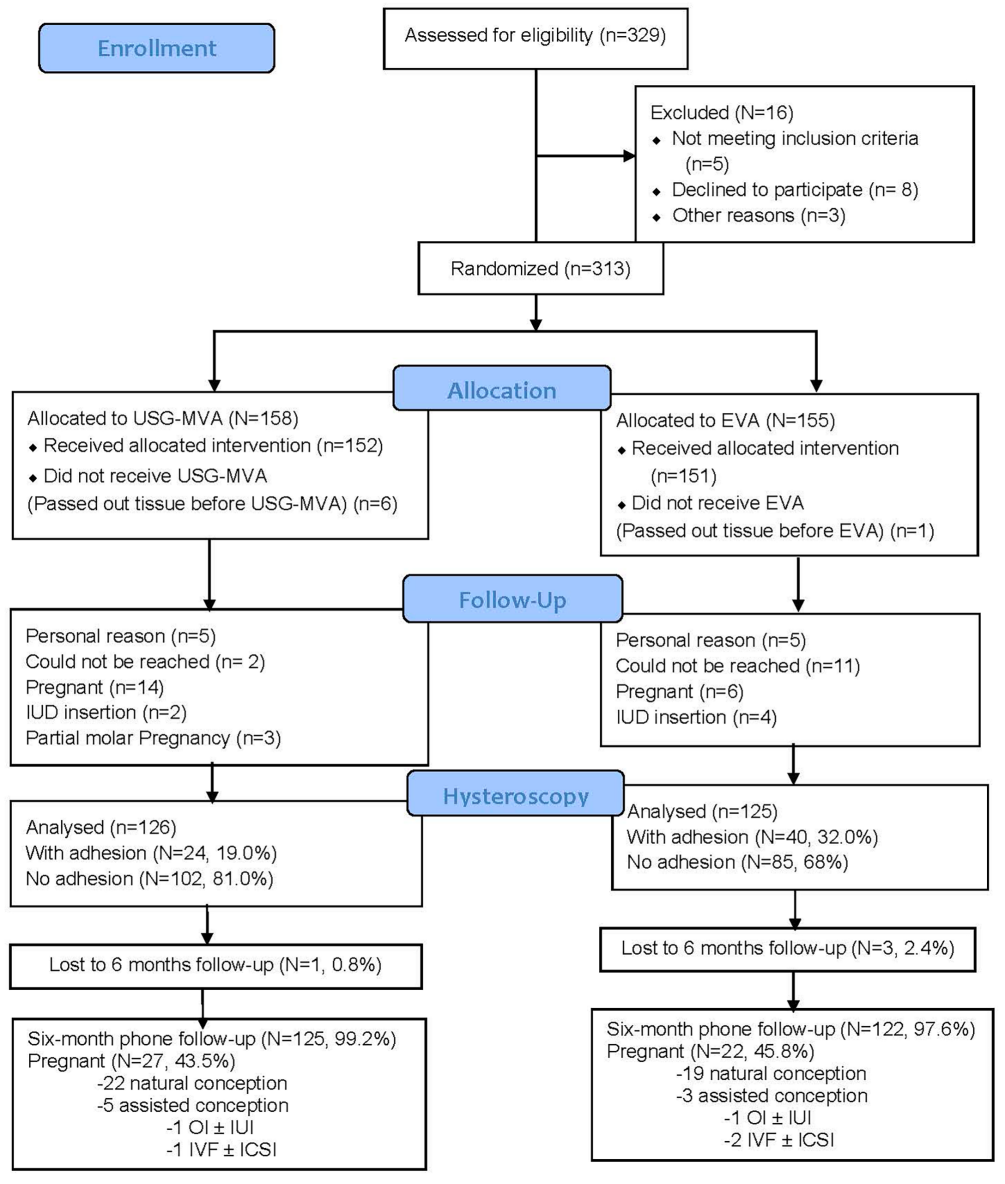
CONSORT flowchart of patients undergone USG-MVA and EVA for the incidence of intrauterine adhesion

A CONSORT flowchart to present the participant flow is shown in Fig. 1. In summary, a total of 251 patients came back and completed the hysteroscopic assessment, 126 from the USG-MVA group, 125 from the EVA group. The baseline characteristics of the patients from two groups are compared and found no significant differences (Table 1).

**Table 1:** Baseline characteristics in women between USG-MVA and EVA group

**Characteristic**	USG-MVA (n = 126)	EVA (n = 125)	*P*	
Baseline Characteristic				
Age (years)	36 (32–39)	36 (32–39)	0.93	
Body Mass Index (kg/m^2^)	22.2 (20.6–24.4)	22.5 (20.5–24.7)	0.96	
Gravida			0.31	
1	27 (21.4%)	25 (20.0%)		
2	43 (34.1%)	33 (26.4%)		
≥ 3	56 (44.4%)	67 (53.6%)		
Previous uterine surgery			0.35	
No	72 (57.1%)	64 (51.2%)		
Yes	54 (42.9%)	61 (48.8%)		
Previous pregnancy loss			0.76	
None	58 (46.0%)	54 (43.2%)		
1	31 (24.6%)	34 (27.2%)		
2	24 (19.0%)	20 (16.0%)		
> or more than 3	13 (10.3%)	17 (13.6%)		
Gestation at Presentation (days)	70 (63–78)	70 (63–81)	0.84	
Menstruation Flow			0.28	
Light	9 (7.1%)	16 (12.8%)		
Normal	106 (84.1%)	101 (80.8%)		
Heavy	11 (8.7%)	8 (6.4%)		
Menstruation Regularity			0.27	
Regular	99 (78.6%)	105 (84.0%)		
Irregular	27 (21.4%)	20 (16.0%)		

Data are given as n (%) or median (Interquartile range)

### Primary outcome

Out of the patients who underwent hysteroscopic assessments, a total of 64 individuals from two groups were diagnosed with IUA following USG-MVA or EVA procedures. The incidence of IUA in the USG-MVA group was 19.0% (*n* = 24/126), while in the EVA group it was 32.0% (*n* = 40/125). The difference in incidence between the two groups was found to be statistically significant (*p* = 0.02). EVA group was also noted to have higher hysteroscopic scoring for AFS than USG-MVA group (*p* = 0.02).

### Secondary outcomes

The hysteroscopic assessment was performed successfully on all patients, with no reported complications in both USG-MVA group and EVA group. The details of the hysteroscopic findings for patients with IUA in both the USG-MVA and EVA groups are presented in Table 2. Our analysis revealed no significant difference (*p* = 0.75) between the time to hysteroscopic assessment in those with IUA in the USG-MVA group (10.36 weeks, range 8.07-13.0), and the EVA group (10.57 weeks, range 7.88–16.3). There is also no significant difference (*p* = 0.65) in the timing of hysteroscopy among those with IUA (10.5 weeks, range 7.9–14.6) and those without IUA (11.1 weeks, range 8.3–16.7).

**Table 2:** Hysteroscopic findings and adhesion assessment by AFS scoring classification in USG-MVA and EVA group

**Characteristic**	USG-MVA (n = 24)	EVA (n = 40)	*P*
Time to hysteroscopy (weeks)	10.36 (8.07-13.0)	10.57 (7.88–16.3)	0.75
AFS* assessment in those with adhesions			
Hysteroscopic Score	2.0 (2.0-3.75)	3.0 (2.0–6.0)	0.02
Extent of Cavity Involved			0.08
< one third	22 (91.7%)	27 (67.5%)	
One third to two thirds	2 (8.3%)	12 (30.0%)	
> two thirds	0 (0.0%)	1 (2.5%)	
Type of IUA			0.24
Flimsy	16 (66.7%)	19 (47.5%)	
Flimsy and dense	6 (25.0%)	12 (30.0%)	
Dense	2 (8.3%)	9 (22.5%)	
Menstrual pattern			0.04
Normal	22 (91.7%)	25 (62.5%)	
Hypomenorrhea	2 (8.3%)	14 (35.0%)	
Amenorrhea	0 (0.0%)	1 (2.5%)	
Prognostic classification			0.01
Stage 1 (Mild)	22 (91.7%)	24 (60.0%)	
Stage 2 (Moderate)	2 (8.3%)	16 (40.0%)	
Stage 3 (Severe)	0 (0.0%)	0 (0.0%)	

Data are given as n (%) or median (Interquartile range)

*AFS: American Fertility Society

Among patients with IUA identified during the follow-up hysteroscopy according to the AFS classification, there were significantly (*p* = 0.01) more patients with Stage 1(Mild) IUA in the USG-MVA group (91.7%, *n* = 22/24) when compared to the EVA group (60.0%, *n* = 24/40). At the time of hysteroscopy, the proportion of subjects who experienced hypomenorrhea at hysteroscopy in the USG-MVA group (8.3%, *n* = 2/24) was significantly (*p* = 0.04) lower than that of the EVA group (35.0%, *n* = 14/40). Concerning the presence of IUA among all participants, there is no significant difference for lighter menstrual flow between IUA and non-IUA groups at the time of OPH (*p* = 0.062) and 6 months postoperatively (*p* = 0.191).

Table 3 presents the hysteroscopic findings and adhesion assessment using AFS in women who underwent USG-MVA or EVA, categorised by previous uterine surgery (including caesarean section, hysteroscopic surgery, and curettage). The incidence of IUA in women with previous surgery (*n* = 36/115) and without previous surgery (*n* = 28/136) showed a *p*-value of 0.052, indicating a lack of statistical significance. After adjusting for gestation (days), gravida, women without previous uterine surgeries demonstrated a trend towards a decreased risk of IUA, although this result was not statistically significant (adjusted odds ratio [aOR] = 0.052; 95% CI 0.208–1.208; *p* = 0.124).

**Table 3:** Hysteroscopic findings and adhesion assessment in USG-MVA and EVA group with or without previous uterine surgery

Characteristic	Women with Previous Uterine Surgery		Women with no Previous Uterine Surgery		
	USG-MVA (n = 12)	EVA (n = 24)	*P*	USG-MVA (n = 12)	EVA (n = 16)
Time to hysteroscopy (weeks)	10.8	10	0.37	10.6	10.9
	(7.8–15.1)	(7.9–18.5)		(8.4–15.0)	(7.7–13.6)
AFS* assessment in those with adhesions					
Hysteroscopic Score	2.5 (2.0–3.0)	3 (2.0–6.0)	0.1	2.0 (2.0–4.0)	2 (2.0–6.0)
**Extent of Cavity Involved**			0.35		
< one third	11 (91.7%)	17 (70.8%)		11 (91.7%)	10 (62.5%)
One third to two thirds	1 (8.3%)	6 (25.0%)		1 (8.3%)	6 (37.5%)
> two thirds					
	0 (0.0%)	1 (4.2%)		0 (0.0%)	0 (0.0%)
**Type of IUA**			0.49		
Flimsy	6 (50.0%)	10 (41.7%)		10(83.3%)	9 (56.3%)
Flimsy and dense	5 (41.7%)	8 (33.3%)		1 (8.3%)	4 (25.0%)
Dense	1 (8.3%)	6 (25.0%)		1 (8.3%)	3 (18.8%)
**Menstrual pattern**			0.05		
Normal	12 (100%)	15 (62.5%)		10(83.3%)	10 (62.5%)
Hypomenorrhea	0 (0.0%)	9 (37.5%)		2 (16.7%)	6 (37.5%)
Amenorrhea	0 (0.0%)	0 (0.0%)		0 (0.0%)	0 (0.0%)
**Prognostic classification**			0.12		
Stage 1 (Mild)	11 (91.7%)	15 (62.5%)		11 (91.7%)	9 (56.3%)
Stage 2 (Moderate)	1 (8.3%)	9 (37.5%)		1 (8.3%)	7 (43.8%)
Stage 3 (Severe)	0 (0.0%)	0 (0.0%)		0 (0.0%)	0 (0.0%)

Data are given as n (%) or median (Interquartile range)

*AFS: American Fertility Society

Regarding the occurrence of hypomenorrhea, the proportion of patients in the EVA group (37.5%, *n* = 9/24) who had previously undergone intrauterine surgeries was higher compared to the USG-MVA group (0.0%, *n* = 0/12). However, this difference did not reach statistical significance.

When IUA was found during hysteroscopy, 24 patients from the USG-MVA group had hysteroscopic adhesiolysis at the same time with the use of hysteroscopic scissors to restore the uterine cavity, while 36 patients from the EVA group underwent this procedure. In the EVA group, two patients required further treatment in the operating theatre, one required a second hysteroscopic adhesiolysis in an outpatient setting while one defaulted further follow-up.

At the 6-month follow-up, a total of 126 patients from the USG-MVA group and 125 patients from the EVA group were successfully contacted. Table 4 presents the menstrual and reproductive outcomes for both groups at this time point. There was no statistically significant difference observed in terms of menstrual regularity and menstrual flow between the two groups at 6 months postoperatively.

**Table 4:** Secondary outcomes of women between USG-MVA and EVA groups ± IUA at 6 months postoperatively

Characteristic	USG-MVA		*P*	EVA		*P*
	No Adhesion (N = 102)	Adhesion (N = 24)		No Adhesion (N = 85)	Adhesion (N = 40)	
**Menstruation**						
Regularity			0.76			0.53
Regular	61 (75.3%)	13 (72.2%)		51 (71.8%)	21 (65.6%)	
Irregular	20 (24.7%)	5 (27.8%)		20 (28.2%)	11 (34.4%)	
Flow			0.78			0.23
Light	25 (30.9%)	6 (33.3%)		27 (38.0%)	14 (43.8%)	
Normal	47 (58.0%)	11 (61.1%)		38 (53.5%)	18 (56.3%)	
Heavy	9 (11.1%)	1 (5.6%)		6 (8.5%)	0 (0.0%)	
**Reproductive outcome**						
Contemplation of pregnancy			0.59			0.89
Yes	49 (48.0%)	13 (54.2%)		33 (38.8%)	15 (37.5%)	
No	53 (52.0%)	11 (45.8%)		52 (61.2%)	25 (62.5%)	
Successful conception	21 (21.4%)	6 (26.1%)	0.63	14 (16.5%)	8 (20.0%)	0.63
Time to pregnancy (weeks)	13	10.83	1	13	13	0.35
	(8.67–17.33)	(8.67–19.5)		(4.33–26.0)	(9.75–13)	
Pregnancy outcome			0.33			0.02
Miscarriage	3 (14.3%)	0 (0.0%)		2 (14.3%)	5 (62.5%)	
On-going pregnancy	18 (85.7%)	6 (100%)		12 (85.7%)	3 (37.5%)	

Data are given as n (%) or median (Interquartile range)

Among the patients who expressed a desire for fertility after the procedure, 62 patients (49.2%) in the USG-MVA group and 48 patients (38.4%) in the EVA group reported such intentions. Of these patients, 27 (43.5%) in the USG-MVA group and 22 (45.8%) in the EVA group successfully conceived (see Fig. 1 for further details). There is no significant difference in the pregnancy rates between the two groups. Moreover, there is no statistical difference between natural conception and assisted conceptions and the following variables: no previous miscarriages (*p* = 0.050), parity (*p* = 0.128), IUA occurrence (*p* = 0.410) and age (*p* = 0.979).

However, a significant finding arose among patients who successfully conceived (*p* = 0.031). In the group that underwent EVA and had IUA, there was a significantly higher rate of miscarriages (62.5%) compared to the group that underwent USG-MVA with IUA, where no miscarriages were reported.

## Discussion

IUA is a known complication after uterine surgery and can impair fertility and lead to reproductive and obstetric complications [6]. Surgical evacuation is one of the commonest uterine surgeries performed in the reproductive aged group [18]. There is thus a recognized need to find alternative surgical ways to protect the endometrium against harm caused by the uterine evacuation.

### Key results

Although IUA can still occur after USG-MVA, the rate of IUA in the MVA group is significantly lower than in the EVA group (*p* = 0.02). This finding suggests that USG-MVA may be a preferable surgical option for individuals who wish to preserve their future fertility. Additionally, among patients with IUA identified during follow-up hysteroscopy, the proportion of cases with mild adhesions was significantly higher in the MVA group compared to the EVA group. Furthermore, the incidence of hypomenorrhea observed during hysteroscopy was significantly lower in the USG-MVA group compared to the EVA group. It is important to note that in the EVA group with IUA, there were more miscarriages observed at 6 months post-surgery among those who attempted pregnancy.

Historically, EVA have been commonly used for the removal of products of conception. However, multiple EVA procedures have been associated with an increased risk of IUA, with reported incidences as high as 38% [7, 19] To mitigate this risk, we have recently introduced USG-MVA in our unit as a potentially less harmful alternative. The incorporation of USG during the procedure enhances safety and improves the completeness of the removal, thereby reducing the likelihood of complications. The use of USG makes the procedure more protective on the endometrium as further evacuation is prevented when ultrasound confirms that the cavity is emptied. This advancement in technology has provided a more precise and controlled approach to the management of first-trimester miscarriage.

However, up till now, there is limited literature to describe the incidence of IUA after MVA. IUA incidence after MVA could only be found in, small case series and observational studies. The rate of IUA from MVA ranges from 6.3 to 16.3% after the treatment of abortion [20, 21]. Previous studies conducted by Dalton et al. identified three cases of symptomatic IUA following MVA [17] and Gilman et al. reported an incidence of 6.3% [20]. Godoy et al. reported an incidence of 16.3% of IUA following MVA for abortion treatment, However, it is important to note that these previous studies on MVA were conducted without the use USG, abortion cases and were based on small number of cases.

This study is the largest RCT to determine on the incidence of IUA in women performing USG-MVA for first-trimester miscarriages. Compared with other studies, the rate of IUA from both USG-MVA and EVA in our study appears to be slightly higher as we have included all flimsy adhesion including lateral and marginal adhesions that may be often missed during hysteroscopic adhesion in earlier reported studies if not carefully examined [22, 23]. The majority of studies included in the previous meta-analysis that examined the prevalence of IUA after EVA were conducted in European and Western countries [10]. Another postulated reason may be due to racial differences. A previous RCT conducted specifically in the Chinese population reported an incidence of IUA after EVA of approximately 30% [24], which is in line with the findings of our study (32%). Moreover, our hysteroscopy was performed 6–20 weeks after the initial surgery and thus may have included some IUA that may be resolved if hysteroscopy was performed later.

### Strengths

Use of hysteroscopy, the gold standard, to assess the rate of IUA is another strength of our study. IUA can be also diagnosed by using hysterosalpingogram, doppler studies or 3D ultrasound [22]. The use of different assessment modalities could potentially explain the heterogeneity in the reported incidence rate of IUA after MVA and EVA. Whilst hysteroscopy is the gold standard assessment for diagnosing IUA, their type and severity can be subjective and therefore biased. The use of a standardised AFS classification of IUA during our hysteroscopic assessment lowered bias [13, 22]. Also, the same group of qualified gynaecologists who performed all hysteroscopic assessments were blinded to the women’s initial surgical procedure performed for the first-trimester miscarriage in our study.

### Limitations

Traditionally, MVA has been performed without USG. Our unit has added USG during MVA as it has been previously shown to have a significant reduction on the need to repeat evacuation and blood loss during the operation [25]. USG allows visualisation of the catheter placement in the uterus, ensures complete evacuation and also minimises the discomfort by keeping the number of passes of the suction catheter to a minimum [26]. We therefore postulated the use of USG also has a theoretical advantage of reducing unnecessary endometrial damage which may explain the lower adhesion rate undergoing USG-MVA in our present study. Performing USG-MVA, as opposed to a blind MVA procedure, however, requires additional equipment and staff with appropriate training.

In this study, a comparison of our USG-MVA with traditional EVA performed in the operation theatre without USG was made. There is no direct comparison between MVA and EVA with USG. In our hospital, we routinely perform EVA without USG, we opted for a more pragmatic protocol design, rather than introducing USG at the time of EVA. Further studies are required to investigate the impact of USG during the EVA process and the incidence of IUA. Moreover, curettage is not performed during USG-MVA. Whereas during EVA, some doctors may opt to perform gentle curettage to assess the completeness of uterine evacuation. This difference in practice may introduce some bias in the comparison between the two procedures and maybe the cause of IUA.

To accommodate the challenges posed by the COVID-19 period, where hysteroscopic sessions were limited due to a shortage of manpower, the timing of hysteroscopy was extended to a range of 6 weeks to 20 weeks. We acknowledge that the variation in assessment time frames could potentially influence the detection of IUA. However, our analysis showed no statistically significant difference in the timing of hysteroscopic assessment between the USG-MVA and EVA groups nor in those with or without IUA.

Moreover, we only followed up with our patients up to 6 months after the initial operation and thus longer menstrual, fertility and obstetric complications arising from IUA were not assessed.

### Future research

More and more operations are increasingly being moved from the operating room to an ambulatory setting to cut costs due to the rising costs of healthcare procedures. One of these procedures, the USG-MVA, can be done in an outpatient setting, saving money on the operating room and general anaesthesia. Also, it increases the comfort and autonomy of the patient [4, 27]. It is necessary to have more cost-effectiveness analyses in order to determine if USG-MVA should be widely used in the healthcare system.

## Conclusions

IUAs are still a possible complication of USG-MVA, even in the absence of sharp curettage. Therefore, patients should be counselled about this risk when discussing treatment options. However, USG-MVA appears to be associated with a lower incidence of IUA postoperatively at 6–20 weeks with less menstrual disturbance when compared with EVA. It should be offered as an alternative surgical option to EVA during the treatment of first-trimester miscarriage.

## Data Availability

The de-identified data sets used and / or analysed during the current study are archived in the Department of Obstetrics and Gynaecology, The Chinese University of Hong Kong. They are available from the corresponding author on reasonable request, subject to IRB review.

## Abbreviations

AFS: American Fertility Society
EVA: Electric vacuum aspiration
IUA: Intrauterine adhesion
MVA: Manual vacuum aspiration
USG-MVA: Ultrasound guidance-manual vacuum aspiration
USG: Ultrasound guidance
